# How can we study reasoning in the brain?

**DOI:** 10.3389/fnhum.2015.00222

**Published:** 2015-04-24

**Authors:** David Papo

**Affiliations:** GISC and Laboratory of Biological Networks, Center for Biomedical Technology, Universidad Politécnica de MadridMadrid, Spain

**Keywords:** cognitive neuroscience, reasoning, scaling, non-stationarity, non-ergodicity, characteristic scales, observation time, resting brain activity

## Abstract

The brain did not develop a dedicated device for reasoning. This fact bears dramatic consequences. While for perceptuo-motor functions neural activity is shaped by the input's statistical properties, and processing is carried out at high speed in hardwired spatially segregated modules, in reasoning, neural activity is driven by internal dynamics and processing times, stages, and functional brain geometry are largely unconstrained *a priori*. Here, it is shown that the complex properties of spontaneous activity, which can be ignored in a short-lived event-related world, become prominent at the long time scales of certain forms of reasoning. It is argued that the neural correlates of reasoning should in fact be defined in terms of non-trivial generic properties of spontaneous brain activity, and that this implies resorting to concepts, analytical tools, and ways of designing experiments that are as yet non-standard in cognitive neuroscience. The implications in terms of models of brain activity, shape of the neural correlates, methods of data analysis, observability of the phenomenon, and experimental designs are discussed.

## Introduction

Consider an individual trying to solve a problem and reasoning for 10 min before attaining a solution. Take the middle 5 min. Clearly, though containing no behaviorally salient event, these 5 min represent a genuine, indeed rather general, instance of reasoning. What do we know about the brain regime far from its conclusion? Can we use this regime to predict a solution, and a solution to retrodict this regime?

Here, I concentrate on a form of reasoning, of which the above scenario constitutes an example, which can broadly be defined as “thinking in which there is a conscious intent to reach a conclusion and in which methods are used that are logically justified” (Moshman, 1995), with no a priori assumption on the type of reasoning process that may take place during it. It is argued that finding the *generic* properties of this form of reasoning entails addressing the following fundamental issues: What are reasoning's temporal and spatial scales? When is a given observation time sufficient? How should we integrate the information contained in various reasoning episodes?

## A mini literature review

The neural correlates of reasoning have traditionally been expressed in terms of brain spatial coordinates. Early neuropsychological work viewed reasoning as emerging from global brain processing (Gloning and Hoff, 1969), consistent with evidence indicating that it is negatively affected by diffuse brain damage (Lezak, 1995). Neuroimaging studies have framed the neural correlates of reasoning in terms of local functionally specialized brain activity, either by taking a normative approach to reasoning (Goel et al., 1997, 1998; Osherson et al., 1998; Parsons and Osherson, 2001; Noveck et al., 2004; Prado et al., 2011), or by fractionating it into sub-component processes (Houdé et al., 2001; Acuna et al., 2002; Kroger et al., 2002; Reverberi et al., 2012). The results often lack specificity to reasoning (Papo et al., 2007). Most importantly, these investigations provide a static characterization of reasoning.

The neuroimaging literature mostly focused on short-term and normative forms of reasoning (Prado et al., 2011; Bonnefond et al., 2013, 2014). This minimizes variability in reasoning episode length and allows segmenting reasoning episodes into separable chunks, but does that at the price of limitations in the phenomenology and ecologic value of its stimuli. Some neuroimaging (Luo et al., 2004; Subramaniam et al., 2008) and electrophysiological (Jung-Beeman et al., 2004; Mai et al., 2004; Kounios et al., 2006, 2008; Lang et al., 2006; Bowden and Jung-Beeman, 2007; Qiu et al., 2008; Sandkühler and Bhattacharya, 2008; Sheth et al., 2008) studies examined more ecological forms of reasoning, viz. insight problems (Knoblich et al., 1999). However, even electrophysiological studies, despite optimal temporal resolution, adopted an event-related perspective, concentrating on activity occurring a few seconds before insight emergence, which only documents the *outcome* of the reasoning process, not the process itself.

Event-related neural activity associated with the solution of riddles with insight was found to be related to properties of preceding resting activity (Kounios et al., 2006, 2008). These studies had the remarkable merit of using spontaneous brain activity to characterize reasoning, but in essence provided a comparative statics description. Although some behavioral studies treated reasoning as a dynamical process (Stephen et al., 2009), a comparable neurophysiological characterization is still incomplete.

## The problem(s) with reasoning

The generalized form of reasoning considered in this study comes in episodes offering scant behaviorally salient events with no characteristic temporal length. Each episode is a non-reproducible instance, as a reasoning task can be carried out in multiple ways. Brain activity associated with reasoning is not event-related, and many neurophysiological processes interact in a wide range of spatial and temporal scales.

These phenomena can all be traced back to a basic fact: the brain did not develop a dedicated device for reasoning. Hardwired partially segregated modules ensure that perceptuo-motor functions are carried out at great speed, with stereotyped duration and time-varying profile, and identifiable stages, largely determined by input statistical properties. Reasoning, on the contrary, is associated with an internally-driven dynamics: processing times and stages, and functional brain geometry are largely unconstrained.

Considering these extraordinary challenges, can we still find general reasoning properties, over and above specific task demands and individual differences? What sort of process is reasoning in its general form? Is it a series of simpler reasoning cycles? Can we segment it into stages? What are the best neural variables and tools to make these properties observable?

## Characterizing the reasoning process

Robust characterizations of reasoning should incorporate properties consistently appearing across different subjects and in different periods of time, and select analytical tools accordingly. For instance, perceptual response sensitivity to incoming signals, stability against noise, and minimal dependence on initial conditions favor tools capturing transient dynamics, which naturally reproduce these properties under appropriate conditions, over tools handling asymptotic activity, which fail to do so (Rabinovich et al., 2008).

Reasoning's relative instability and inefficiency suggest that optimal circuitry may need constant reconstruction and protection from interference, summoning protracted support of energetically costly long-range communications. Reasoning may be a sort of resonant regime, where functional efficiency would be achieved with specific, though unstable, spatio-temporal patterns. This suggests that reasoning should be studied with tools which can describe spatially-extended dynamic transients and can quantify information transfer and the corresponding energetic cost.

### Reasoning dynamics

Each cognitive process can be translated in dynamical terms and corresponding aspects of neural activity.

Perceptual processes are relaxational, quasi-stereotyped short duration processes. The brain can *prima facie* be modeled as an excitable medium: perturbations above a threshold induce a dynamical cycle before the system reverts to its initial silent state.

Learning too is a relaxational process. Following a gradient dynamics, the brain incorporates the environment's statistical relationships by representing them in terms of its functional connectivity (Sporns et al., 2000). Cycles can be of much longer duration and non-trivial shape than perceptual ones. No single instant summarizes the entire process, and the dynamics consists of fluctuations much shorter than the whole process.

Reasoning may not be purely relaxational. As in the case of learning, no instant summarizes the whole dynamics but, contrary to learning, there is no clear gradient. Neural activity is an out-of-equilibrium endogenously modulated spontaneous brain activity. Its phenomenology is considerably more complex than the equilibrium event-related short time-scale one of perception or the gradient-driven regression to equilibrium dynamics of learning.

To study reasoning, one should therefore first consider properties of spontaneous activity that are *generic* (i.e., that hold for almost all conditions) at long time scales and then see how these properties are modulated during reasoning (Papo, 2014a).

### The starting point: spontaneous brain activity

When observed long enough, brain fluctuations appear to be characterized by structured patterns (Kenet et al., 2003). The temporal sequence with which these patterns are re-edited across the cortical space also appears to have non-random structure (Beggs and Plenz, 2003, 2004; Cossart et al., 2003; Ikegaya et al., 2004; Dragoi and Tonegawa, 2011; Betzel et al., 2012). The structure with which these fluctuations appear can be described in the same way one would describe an object, characterizing its component parts, the relationships between them, and the way one can inspect it. For instance, if we think of brain fluctuations as the steps of a random walker, one can describe the *phase space*, i.e., the space of all states attainable by the system's dynamics, but also of traveled distances, times to reach a given target and memory of previous steps.

In the equilibrium world of perceptual scientists, brain steps are Gaussian distributed, and memory of past steps is lost so rapidly that no structure is apparent when considering the time course of activity. Spontaneous activity has no evident temporal structure and can be treated as a null state to which the brain reverts in the absence of stimulation.

At the long time scales of reasoning, the random walker takes steps from a non-Gaussian distribution. Like a fractal object, it displays similar properties at all scales (Novikov et al., 1997; Linkenkaer-Hansen et al., 2001; Gong et al., 2002; Freeman et al., 2003; Stam and de Bruin, 2004; Expert et al., 2010; van de Ville et al., 2010; Fraiman and Chialvo, 2012). While self-similarity may not be exact (Suckling et al., 2009; Zilber et al., 2012), these scaling patterns indicate that activity at different temporal scales is characterized by non-trivial relationships between them (Bacry et al., 2001; Friedrich et al., 2011; Papo, 2013b). Not all regions of the phase space are equally visited, with some taking an extremely long time to be reached (Bianco et al., 2007). Transitions from one region to the other depend on past history of the dynamics (Gilboa et al., 2005). Memory of past steps decays so slowly that the time it takes two time-points to totally decorrelate may diverge, so that a characteristic time ceases to exist (Grigolini et al., 1999; Fairhall et al., 2001; Gilboa et al., 2005; Lundstrom et al., 2008). Temporal correlations are not stationary, but time-dependent (Bianco et al., 2007). If, rather than an ordinary watch, one measured time with a watch ticking at every step taken by the walker, the passage of time would appear to be highly irregular and clustered, alternating between relatively quiet phases and more turbulent ones (Gong et al., 2007; Allegrini et al., 2010).

The temporal structure can be used to define landmarks within time-windows where no behaviorally salient event occurs. This can be done by identifying segments that can be considered stationary (Kaplan et al., 2005). The distribution of these segments' durations and their correlations and specific sequences may help clarify whether reasoning far away from both problem presentation and solution is merely a repetition of simple cycles seen in more controlled forms of reasoning, or is of a qualitatively different nature, and if so, may help determine the time scales at which simpler cycles are reedited.

To fully describe the phase space, one needs to consider that the brain as a whole consists of a great number of local random walkers. Local walkers interact to form transient patterns of connectivity. These patterns can be endowed with topological properties at all spatial scales by resorting to complex networks theory (Bullmore and Sporns, 2009). Eventually, one deals with an abstract structure consisting of spatial patterns endowed with topological properties, the temporal evolution of which displays the complex properties described above.

Overall, the space in which the random walker turns out to live, and which reflects the brain's dynamical repertoire, can be represented as a complex spatio-temporal structure (Zaslavsky, 2002). This structure can be described in terms of symmetries and universal properties, which are robust with respect to the nature of microscopic details, by resorting to a variety of methods, e.g., algebraic and differential topology, renormalization group methods etc. (Lesne, 2008; Petri et al., 2014). Using these methods it is possible (1) to partition the phase space, (2) to identify dynamical pathways leading to specific regions of this space, and (3) to relate descriptions of the same brain at different scales and of different brains exhibiting the same large-scale behavior (Lesne, 2008).

### From spontaneous activity to reasoning

Cognitive processes can be thought of as selections and orchestrations of cortical states already present in spontaneous activity (Kenet et al., 2003; Fiser et al., 2004; Luczak et al., 2009). Each process reveals a specific part of the phase space, and can be associated with its own topological properties and symmetries, and characteristic kinematics, memory, aging properties, degree of ergodicity, and internal clock (Papo, 2014a). For example, different conditions under which subjects carried out a reasoning task were shown to modulate the scaling regime of fluctuations of the corresponding brain activity (Buiatti et al., 2007), suggesting that reasoning may modulate not brain activity's amplitude but its functional form (Papo, 2014a), e.g., by forcing the system's stationary distribution to equal a target one. These modulations may correspond to cross-overs between universality classes, resulting from transitions between different dynamical regimes (Burov and Barkai, 2008).

The statistics of fluctuations can be used to study insight and to evaluate whether insight occurrence can be predicted. The sudden onset of insight may be thought of as an extreme event comparable to earthquakes, financial crashes, or epileptic seizures (Contoyiannis and Eftaxias, 2008; Osorio et al., 2010), e.g., as a rupture phenomenon, and the route to it as a long charging process, with nested hierarchical “earthquakes.” The probability distribution of fluctuations gives an estimate of the likelihood of the occurrence of such events: for a Gaussian distribution, extreme events are exponentially rare. However, for non-Gaussian distributions, such events do occur with non-zero probability. It is tempting to conjecture that, in analogy with results of studies of these phenomena, insight onset may be predicted by monitoring changes in anomalous diffusion parameters (Contoyiannis and Eftaxias, 2008), Gaussianity (Manshour et al., 2009), or fractal spectrum complexity (de Arcangelis and Herrmann, 1989; Kapiris et al., 2004).

#### Assessing reasoning: from dynamics to thermodynamics and information

Considering the functions reasoning fulfills and the constraints the brain faces while performing it can shed light on ways in which brain fluctuations can help quantify how the brain carries out reasoning.

Reasoning, as other cognitive processes, e.g., memory recall (Rhodes and Turvey, 2007; Baronchelli and Radicchi, 2013), can be represented as a search process similar to that of animals foraging in an unknown environment (Viswanathan et al., 2011). This search process can be characterized in terms of random walks (Shlesinger et al., 1993; Codling et al., 2008; Lomholt et al., 2008; Bénichou et al., 2011). Importantly, the statistics of random steps and their correlations indicate the extent to which a given trajectory optimizes search, given the characteristics of the explored space and the resources available to the individual (Bénichou et al., 2011). Such a characterisation would allow assessing in a context-specific way the quality of both the reasoning and the “reasoned.” That behavioral aspects of human cognition (Rhodes and Turvey, 2007; Baronchelli and Radicchi, 2013) and brain activity both show non-Gaussian, heavy-tailed distributions might indicate search optimality (Lomholt et al., 2008; Humphries et al., 2012). However, because these properties are generic in spontaneous activity, reasoning's quality can only be described in terms of its modulations, and finding the neural property and spatial scale showing such scaling modulations are the crucial steps.

Because it lacks a hardwired structure, reasoning faces both a stability and an energetic problem. Fluctuation dynamics can help address the first issue, but may not be sufficient *per se* to address the second. While a graph theoretical representation of functional brain activity may provide indications as to the ways the brain tackles both problems (Bullmore and Sporns, 2012; Papo et al., 2014), a direct characterization can be achieved by considering the brain as a very complex engine and by characterizing its thermodynamics. Crucially, thermodynamics can be deduced from dynamics (Sekimoto, 1998). Such a characterisation could be used to quantify variations in thermodynamic variables such as free energy, entropy, or temperature (Papo, 2013a) during a reasoning task, but also possible transitions in some other property of neural activity, for particular values of these variables. For instance, a suitably modified equilibrium temperature accounting for the non-equilibrium nature of brain activity (Cugliandolo, 2011) can quantify deviations of each spatio-temporal scale from equilibrium, entropy production, etc. (Papo, 2014b).

Finally, one may want to quantify reasoning in terms of the information created, erased, and transferred during its execution. Simple fluctuations can be thought of as letters of an alphabet, fluctuation complexes as words, and the reasoning process represented as a network traffic regulation problem. Characterizing traffic regulation and phenomena such as overload or jamming may involve using information-theoretical tools and complex network theory and understanding the interplay between the underlying network's topology, the dynamics of information packets and the shape of fluctuation distributions (DeDeo and Krakauer, 2012; Delvenne et al., 2013; Lambiotte et al., 2013). Although only causal information (Shalizi and Moore, 2003) may directly serve reasoning purposes, the total information encoded in the network may describe the noise-control mechanisms indirectly optimizing it. Interestingly, non-equilibrium systems such as the brain, information, and thermodynamics can be thought of as the opposite side of the same coin (Parrondo et al., 2015). Ultimately, the information content of reasoning-related neural activity could be extracted from its dynamics, via thermodynamics.

## From theory to experiment

### Observing reasoning

Reasoning is a difficult phenomenon to observe: tasks can be executed in more than one-way, each possibly corresponding to a neural phase space with convoluted geometry and the processes involved in reasoning may evolve over time-scales exceeding those typical of laboratory testing.

Proper observation of a given process requires that the *observation time* be much larger than any scale in the system. A process is observable if it has a finite ratio between the characteristic time of the independent variable and the length of the available time series (Reiner, 1964). Factors including long-term memory, aging and weak ergodicity breaking may result in a diverging ratio (Rebenshtok and Barkai, 2007).

The observation time should also be much larger than the time needed to visit the neural phase space. The time needed to explore this space may far exceed the typical reasoning episode duration. Cognitive neuroscientists observe phenomena through experiments where subjects typically carry out given tasks a large number of times, assumed to be independent realizations of the same observable, and to adequately sample the phase space of task-related brain activity. However, in the presence of complex fluctuations, trials may not *self-average*, i.e., dispersion would not vanish even for an infinite number of trials (Aharony and Harris, 1996). Thus, trials may explore different aspects of the space of available strategies and may therefore improve phase space exploration rather than the signal-to-noise ratio (Ghosh et al., 2007).

### Experimental implications

Reasoning's characteristics, particularly its lack of characteristic temporal duration, have implications at various levels. First, episodes cannot be compared in an event-related fashion. Second, defining reliable neural correlates of reasoning requires defining its characteristic temporal scales. Third, measures of brain activity should be invariant with respect to overall duration. Scaling exponents, data collapse and universality of fluctuations statistics (Bramwell et al., 1998; Bhattacharya, 2009; Friedman et al., 2012), or explicit evolution equations for the particle's momenta and for the cross-scale fluctuation probabilities (Friedrich et al., 2011) can be retrieved from data and applied to unevenly lengthen trials. Thermodynamic quantities such as free energy or temperature can also be estimated for stochastic trajectories over finite time durations (Ruelle, 1978; Beck and Schlögl, 1997; Canessa, 2000; Olemskoi and Kokhan, 2006; Papo, 2014b). In all cases, the reconstruction of the underlying dynamics improves with the recording device's resolution.

Reasoning presents a dilemma between ensuring complete phase space exploration, which may require extremely long trials, and signal stationarity, which is guaranteed only for time scales much shorter than the reasoning episodes' duration. At fast time scales, the window in which relevant quantities are calculated should not introduce spurious time scales, filtering out genuine ones. Altogether, reasoning's inherently unstable nature suggests that describing it may boil down to characterizing non-stationarities and their aetiologies.

Reasoning tasks may be so difficult that only few participants manage to produce solutions within a reasonable time. This represents a shortcoming when trials are considered as independent and identically distributed, as the signal-to-noise ratio improves with the square root of the number of trials. Smoothing response times is a frequent strategy to obviate this problem, but limits or distorts the reasoning process. Furthermore, however many, short trials may insufficiently explore the phase space. Designs with few long trials may express richer spatio-temporal brain dynamics than many short ones of equivalent overall length.

Finally, while observed scaling properties may help us understand whether insight is *predictable*, i.e., whether it is an outlier or it is generated by the same distribution producing anonymous events, predicting insight onset in real data appears to be a challenging task, as reasoning episodes are various orders of magnitude shorter than earthquake, financial, or epilepsy time series (Sornette, 2002).

## Conclusions

Reasoning elicits an exceptionally rich repertoire of otherwise unexpressed neural properties. Its neural correlates are therefore as helpful to neuroscientists, who are compelled to consider hitherto neglected brain properties, as they are to psychologists who strive to understand its underlying processes.

Defining general and robust mechanistic properties of healthy and dysfunctional reasoning will require as yet non-standard brain metrics, experimental designs, and analytical tools, and may ultimately help us understand and fine-tune the action of brain enhancers.

### Conflict of interest statement

The author declares that the research was conducted in the absence of any commercial or financial relationships that could be construed as a potential conflict of interest.
